# Output-Mode Transitions Are Controlled by Prolonged Inactivation of Sodium Channels in Pyramidal Neurons of Subiculum

**DOI:** 10.1371/journal.pbio.0030175

**Published:** 2005-05-03

**Authors:** Donald C Cooper, Sungkwon Chung, Nelson Spruston

**Affiliations:** **1**Department of Neurobiology and Physiology, Institute for NeuroscienceNorthwestern University, Evanston, IllinoisUnited States of America; **2**Department of Psychiatry, University of Texas Southwestern MedicalDallas, TexasUnited States of America; **3**Department of Physiology, Sungkyunkwan University School of MedicineSuwanSouth Korea; Johns Hopkins University School of MedicineUnited States of America

## Abstract

Transitions between different behavioral states, such as sleep or wakefulness, quiescence or attentiveness, occur in part through transitions from action potential bursting to single spiking. Cortical activity, for example, is determined in large part by the spike output mode from the thalamus, which is controlled by the gating of low-voltage–activated calcium channels. In the subiculum—the major output of the hippocampus—transitions occur from bursting in the delta-frequency band to single spiking in the theta-frequency band. We show here that these transitions are influenced strongly by the inactivation kinetics of voltage-gated sodium channels. Prolonged inactivation of sodium channels is responsible for an activity-dependent switch from bursting to single spiking, constituting a novel mechanism through which network dynamics are controlled by ion channel gating.

## Abstract 2

Prolonged inactivation of sodium channels represents a mechanism through which network dynamics may be controlled by ion channel gating properties.

## Introduction

In thalamus, high frequency bursting of action potentials has been suggested to maximize stimulus onset detection, while prolonged depolarizations switch the thalamic output mode to tonic firing (single spiking), yielding a more linear reflection of the excitatory synaptic drive [1]. Relatively long periods of quiescence followed by bursts are observed during wakefulness, when they may powerfully activate the cortex in response to salient sensory stimuli [1–6]. By contrast, continuous, oscillatory bursting of thalamic relay neurons is a hallmark of some stages of sleep and is thought to interfere with sensory stimulation of the cortex during sleep [7]. Output-mode switching is likely to be an important general mechanism through which neurons in other brain regions code for different stimulus features [8]. Understanding the mechanisms responsible for transitions between bursting and single-spiking output modes is an important goal, because it may provide insight not only into stimulus feature coding, but also how brain-state transitions influence attentional states.

Lying at the interface between the hippocampus and the entorhinal cortex, the subiculum provides the major output from the hippocampus to a number of brain structures [9]. By analogy to the thalamus, the subiculum can be viewed as a relay structure that transmits information from the hippocampal formation to subcortical and cortical areas, such as the prefrontal and entorhinal cortex. In humans and rodents alike, the subiculum consists of intrinsically bursting neurons that exhibit transitions from bursting to single-spike firing [6,10–12]. Unlike thalamic neurons, however, where bursting is voltage dependent and driven by inactivating, low-voltage–activated (T-type) Ca^2+^ channels [13,14], bursting in the subiculum is voltage independent and driven primarily by non-inactivating, high-voltage–activated Ca^2+^ tail currents [15]. Consequently, depolarization alone is incapable of switching the output from bursting to single-spike firing in subicular neurons [12,16]. Given this difference in the mechanism of bursting, we hypothesized that the mechanism for output-mode switching in the subiculum would be fundamentally different from that of the thalamus. We endeavored to determine the mechanism for subicular output-mode transitions, because it may provide general insight into how neurons conditionally regulate their output, and further our goal of understanding how the subiculum integrates hippocampal information during normal function and disease.

## Results

**Figure 1:**
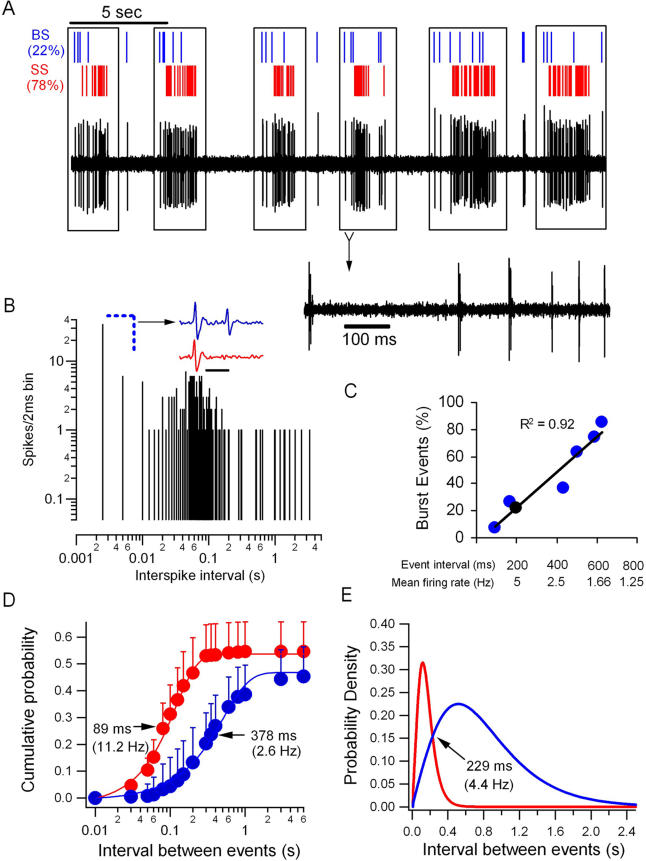
In Vivo Action Potential Output Mode Transition in Bursting Dorsal Subicular Neurons (A) Representative 30-s trace shows six epochs of bursting-to-single spiking output mode transition. Each epoch began with bursts that switched to single spikes. Bursts (BS, blue) and single spike (SS, red) events were extracted from the raw data (black trace) for each cell, and the percentages of spike events (burst or single spikes) were determined. For this cell, 22% of the total events were bursts, while 78% of events were single spikes. The lower inset depicts an expansion from one epoch showing the transition from bursting to single spiking. (B) Interspike interval histogram for the neuron in (A) shows a bimodal distribution of short intervals of less than 8 ms (blue dashed lines show burst intervals), and of longer intervals between 40 and 200 ms. The arrow points to an expanded burst (blue) and single spike (red) extracellular waveform (scale bar, 2 ms). (C) A strong positive correlation was observed between the percentage of burst events and the average event interval (*n* = 7). The filled black circle shows the relative position of the cell from (A) and (B). (D) The cumulative probability plot shows the probabilities of total burst (45%) and single spike (55%) events across inter-event intervals for all cells (*n* = 7). A sigmoidal function was used to fit the data (half max SS [red] = 89 ms, BS [blue] = 378 ms). (E) Probability density plot shows the fractional probability change across the inter-event interval. The peak probability density for bursting (blue) was longer than for single spiking (red) events; the arrow indicates the interval beyond which the probability of bursting exceeds that of single spiking.

Spontaneous extracellular single-unit activity and population field recordings from the dorsal subiculum of anesthetized adult rats (6–7 wk old) exhibited epochs of spontaneous firing with dominant delta (1–3 Hz) and theta (5–10 Hz) frequency bands (Figures 1 and S1). Each epoch of activity began with bursting and then shifted to predominantly single spiking in all subicular neurons recorded (*n =* 7; Figure 1A). Interspike interval histograms of extracellular units showed a bimodal distribution, with a narrow interspike interval peak associated with the high-frequency bursts of two or three action potentials at intervals of 2–8 ms, and a broader second peak consisting of primarily single spiking at intervals of 40–200 ms (Figure 1B and 1E). There was a positive correlation between the inter-event interval and the probability of bursting, such that cells with a higher rate of spontaneous action potential firing were least likely to burst (Figure 1C). The average cumulative probability plot of pre-event silent periods indicates that 55% percent of all events were single spikes (range 14%–92%), while 45% were bursts (range 8%–86%, *n =* 7; Figure 1D). The median interval before a single spike was 89 ms, while the median interval before a burst was 378 ms (Figure 1D), indicating that single-spike and burst event probability is related to the pre-event silent period. To determine how event probability changed as a function of inter-event interval, a probability density plot was obtained by differentiating the cumulative probability curve fit. For intervals less than 229 ms (4.4 Hz) the probability of a single spike was greatest, while for longer intervals (over 229 ms) the probability of a burst was greatest (Figure 1E). Consistent with this result, the average pre-event silent period was significantly longer for bursts (418 ms, 2.4 Hz) than for single spikes (153 ms, 6.5 Hz, *n =* 7; *p* < 0.01). Thus, the transition from bursting to single spiking corresponds to the transition from the delta to the theta frequency band. Two possible mechanisms could account for this result: Either the subicular neurons in vivo burst in response to strong synaptic inputs following long silent intervals and switch to tonic firing as synaptic inputs depress, or intrinsic conductances of the subicular neurons may undergo changes in activation states that determine the output mode during constant synaptic drive.

**Figure 2:**
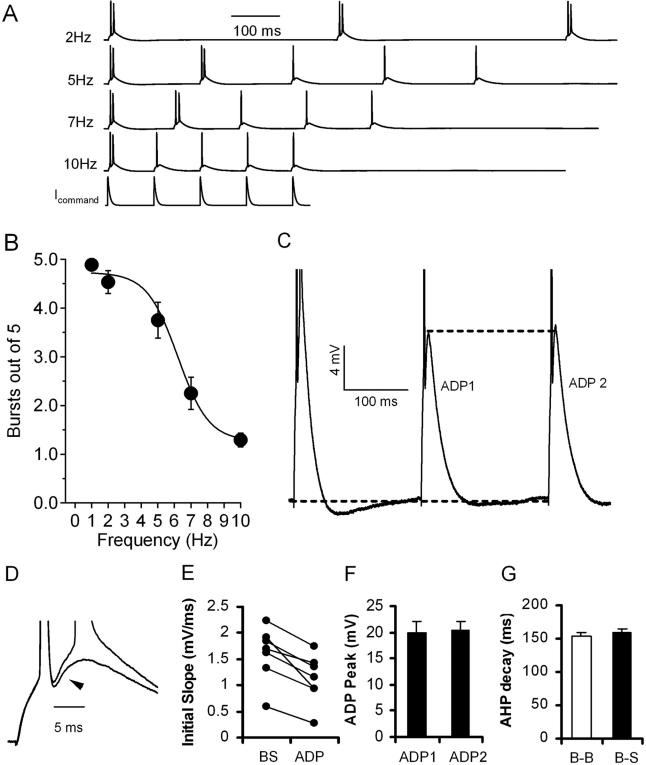
In Vitro Transition from Bursting to Single Spiking Output Is Frequency-Dependent (A) A representative cell shows the typical burst-to-single spike transition in response to current injections of five sEPSCs (τ_decay_ = 6 ms) delivered at frequencies between 2 and 10 Hz. Note: Only the 10 Hz sEPSC is shown. (B) The average number of bursts in response to five sEPSC injections delivered at frequencies of 1–10 Hz (*n* = 17). (C) The representative trace shows the lack of a change between the ADP peaks (ADP1 versus ADP2) that follow a burst (5 Hz) and the complete decay of the AHP before the transition from bursting to single spiking. (D and E) A representative trace (D) and averaged data (E) show that the initial slope of the ADP is decreased during the transition from bursting to single spiking at 5 Hz (*n* = 7). The arrow in (D) points to the beginning of the ADP slope in an overlay of a burst before and after a transition to a single spike at 5 Hz. (F) The average peak of the ADP (in the absence of a second spike) does not change during a train (5 Hz; *n* = 4). (G) The average decay of the AHP (time for *V*_m_ to return to rest) is not different between two bursting events (B-B, *n* = 8) and transitions from bursting to single spike events (B-S, *n* = 8) at frequencies of 2–5 Hz.

**Figure 3:**
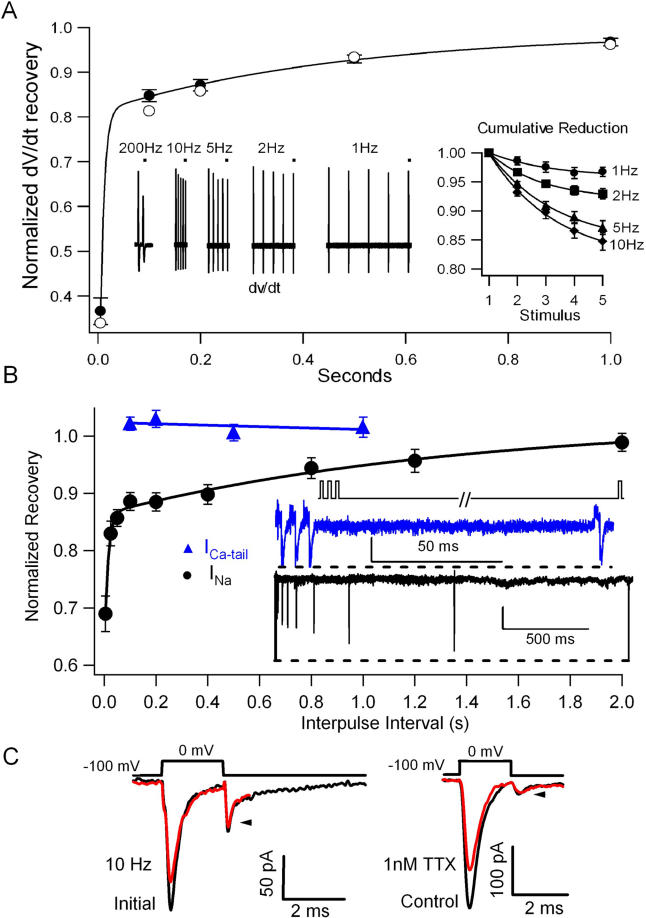
Transitions from Bursting to Single Spiking Output Are Mediated by Slow Recovery from Na^+^ Current Inactivation (A) Filled black circles show the *dV/dt* of the first action potential response to the fifth suprathreshold sEPSC input at frequencies between 1 and 10 Hz (*n* = 14; open circles, 5 mM BAPTA, *n* = 3; recorded at 34 °C). The inset shows a cumulative decrease in the action potential rise rate *(dV/dt)* (τ_1Hz_ = 17,980 ms, τ_2Hz_ = 925 ms, τ_10Hz_ = 250 ms; *n* = 14). (B) Recovery from burst-induced (two or three step pulses [2 ms] from −100 mV to 0 mV) inactivation of Na^+^ current (black, *n* = 11 cell-attached patches) and Ca^2+^ current (blue; *n* = 9 [pooled cell attached, *n* = 4, and nucleated patches, room temperature, *n* = 5]). The inset shows examples of the burst-induced slow inactivation of Na^+^ current (black, vertical scale bar is 50 pA) and the lack of slow inactivation of the Ca^2+^ tail current (blue, vertical scale bar is 5 pA). *I*_Na_ (black) is an overlay of multiple traces, each with an initial burst-like current followed by a single test pulse at different recovery intervals. (C) The left tracing shows a nucleated outside-out patch recording (at room temperature) comparing Na^+^ current in response to a single step pulse (2 ms) in control (black) or 100 ms (10 Hz) after burst conditioning (red). Arrowhead shows the lack of reduction in the *I*_Ca_ (tail). The right tracing shows a reduction of the Na^+^ current without a change in the *I*_Ca_ (tail) after bath application of 1 nM TTX (black, control; red, TTX). Values are reported as mean ± standard error of the mean.

To address this issue, we studied the intrinsic properties of subicular neurons in the acute brain-slice preparation. Patch-clamp recordings were obtained from subicular pyramidal neurons in hippocampal slices prepared from mature rats (Figure S2) [12,15,16]. Neuronal responses to noisy current injections were found to undergo transitions from bursting to single spiking that mimicked the in vivo transitions (Figure S3). Similarly, in response to 5-Hz synaptic stimulation of CA1 afferents to subiculum, the majority of bursting neurons (seven out of eight) exhibited a transition from bursting to single spiking during the train. To determine whether the transition from bursting to single spiking was the result of a postsynaptic, intrinsic property of bursting neurons, we injected simulated excitatory synaptic currents (sEPSCs) that produced simulated excitatory postsynaptic potentials (EPSPs) with rise and decay kinetics similar to synaptically evoked EPSPs [16]. The sEPSCs were injected at frequencies of 1–10 Hz in the presence of blockers of glutamatergic, GABAergic, and muscarinic synaptic receptors (2.5 mM kynurenic acid, 2 μM SR 95531, and 1 μM atropine, respectively) (Figure 2). At frequencies between 1 and 10 Hz, bursting neurons switched from bursting to single spiking (Figure 2A and 2B). This transition was not associated with a progressive change in the peak of the single-spike fast afterdepolarization (ADP1 versus ADP2; Figure 2F), suggesting that a change in the Ca^2+^ tail current driving the ADP was not responsible for the switch from bursting to single spiking. Furthermore, this transition persisted at a frequency (5 Hz) where the post-burst afterhyperpolarization (AHP) had decayed completely (*n* = 8; Figure 2G). The mode switching was associated with a change in the initial slope of the ADP, however, suggesting that the mechanism of bursting is activated within 1 ms of the initial spike repolarization (Figure 2D and 2E). Mode switching was also associated with a frequency-dependent and cumulative reduction in the rate of rise *(dV/dt)* of the initial action potential in a burst (a 17% ± 1% decrease in the peak rate at 10 Hz and a 96% ± 1% recovery at 1 Hz, *n =* 14). Analysis of this reduction in *dV/dt* indicated that the reduction in the process responsible for this change was frequency dependent, cumulative, and slow to recover (Figure 3A). The *dV/dt* recovery to control level was fit by a double exponential (τ_fast_=10.3 ms, τ_slow_=545 ms). The relative contributions of the fast and slow components to the recovery of *dV/dt* were 80% and 20%, respectively. The action potential *dV/dt* is proportional to Na^+^ channel availability [17], so we focused our attention on voltage-gated Na^+^ channel inactivation as a possible mechanism for the transition from bursting to single-spiking mode.

Previous work has shown that recovery from prolonged inactivation of Na^+^ channels in CA1 pyramidal neurons [17–19] exhibits a time course similar to the transition from bursting to single spiking we observed in subiculum. To determine whether this property could be responsible for output mode transition, we measured currents in cell-attached and outside-out nucleated patches obtained from the soma of subicular neurons (see Materials and Methods). In these patches, brief depolarizing voltage commands elicited rapidly inactivating Na^+^ currents (*I*_Na_; τ_inact_ = 0.36 ± 0.024 ms; *n* = 9). We studied recovery from inactivation of *I*_Na_ by using two or three brief (2 ms) depolarizations at 200 Hz to mimic Na^+^ channel inactivation during action potential bursting. Recovery of *I*_Na_ was probed using a test pulse (2 ms) at variable times (10–2000 ms) after the initial burst-like depolarization (Figure 3B). *I*_Na_ recovery from prolonged inactivation was fit by a double exponential (τ_fast_ = 3.7 ms, τ_slow_ = 934 ms). The relative amplitudes of the fast and slow recovery from inactivation of *I*_Na_ were 86% and 14%, respectively. The slow time constant is similar to the time course for mode switching and the slow and cumulative reduction in the *dV/dt* (Figure 3A and 3B), suggesting that Na^+^ channel availability may regulate the transition from bursting to single-spiking (96% Na^+^ channel recovery at 1.2 s for nucleated patches compared to 96% recovery in the action potential *dV/dt* at 1 Hz stimulation).

The Ca^2+^ tail current, shown to be important for the ADP that drives bursting [18], was measured in cell-attached or outside-out nucleated patches in the presence of blockers for voltage-gated Na^+^ and K^+^ channels (500 nM TTX, 5 mM 4-aminopyridine, 30 mM tetraethyl ammonium chloride, and 130 mM internal Cs^+^). The resulting Ca^2+^ current exhibited biexponential deactivation (τ_fast_ = 0.44 ± 0.024 ms, τ_slow_ = 1.65 ± 0.17 ms, voltage stepped from −70 mV to 0 mV, *n =* 7). There was no evidence for either fast or slow inactivation of the Ca^2+^ tail current when repeated at frequencies of 5–10 Hz (Figure 3B). Similarly, outward K^+^ currents contributed the termination of the burst, but voltage-activated K^+^ currents evoked by brief depolarizations did not change when evoked repeatedly at 5–10 Hz (Figure S4).

**Figure 4:**
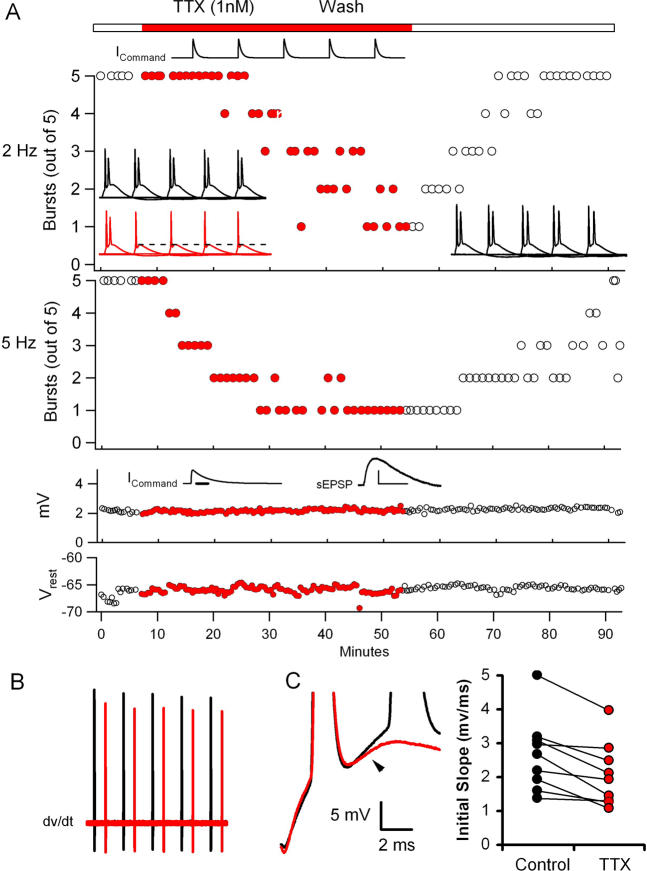
Modest Reduction of Na^+^ Current Induces a Frequency-Dependent Output-Mode Transition from Bursting to Single Spiking (A) The number of bursts in response to a train of five suprathreshold sEPSC currents injections at 2 and 5 Hz delivered every 20 sec before, during (red), and after bath application of 1 nM TTX. The black (left), red, and black (right) insets show overlays of the action potential transition in response to five 2-Hz sEPSC inputs (*I*_Command_) under baseline, TTX (1 nM), and wash conditions, respectively. Note the dashed line in the TTX (red) trace shows the lack of change in the ADP during the stimulus train. The lower panels show no change in a subthreshold sEPSP or resting potential (mV) in response to TTX. The insets show an expanded sEPSC (left; scale bar, 5 ms) and the corresponding sEPSP (right; scale bars, 1 mV vertical, and 20 ms horizontal) that was used to monitor changes in passive properties of the cell. (B) A representative trace showing the reduction in the *dV/dt* of the action potentials before (black) and after 1 nM TTX (red). The average reduction in the first action potential *dV/dt* after 1 nM TTX was 9% ± 2% (*n* = 5). (C) An overlay (left tracing) showing the reduction in the initial slope of the ADP before (black burst) and after (red single spike) 1 nM TTX (All traces in [A], [B], and [C] were taken from the same recording). The graph on the right shows that the low concentrations of TTX (1–5 nM) necessary to induce a switch from bursting to single spiking decrease the initial slope (as illustrated in [C]) of the ADP for a burst (black) and compared to the first transition to a single spike (red) at low frequencies (less than 0.1 Hz) that alone do not influence the burst-single spike transition (ADP reduction = 23% ± 6%, *p* < 0.003, *n* = 9).

To more directly test the hypothesis that prolonged inactivation of *I*_Na_ is responsible for the transition from bursting to single spiking, we used a low concentration of tetrodotoxin (TTX) to block a fraction of Na^+^ channels similar to that removed by prolonged inactivation. We reasoned that if small reductions in *I*_Na_ are capable of inducing a switch from bursting to single spiking, then very low concentrations of TTX should influence the mode transition. Because most subicular neurons switch from bursting to single spiking in response to suprathreshold input spaced 100 ms apart (10 Hz; see Figure 2B), we first determined that 11% ± 2% of *I*_Na_ is unavailable 100 ms after two conditioning pulses mimicking a burst (Figure 3B and 3C). A similar fraction of *I*_Na_ was blocked by 1 nM TTX (16% ± 2%, *n* = 6; Figure 3C). In current-clamp recordings, 1 nM TTX induced a transition from bursting to single spiking at low frequencies (2 Hz), where subicular neurons normally burst reliably (*n* = 5; Figure 4A), and reduced the *dV/dt* of the initial action potential in a burst by 9% ± 2% (*n* = 5; Figure 4B). The ability of 1 nM TTX to accelerate the output-mode transition was completely reversible in most cases and was not associated with a change in the subthreshold response to sEPSCs or a change in the resting potential during recording (Figure 4A). Similar results were obtained at slightly higher concentrations of TTX (2–5 nM); however, at these concentrations, bursting was completely blocked and replaced by single spiking, even at very low frequencies of less than 0.1 Hz (unpublished data). A decrease in the initial slope of the ADP (−23% ± 6%, *n* = 9) was associated with a frequency-independent, TTX-induced mode transition (Figure 4C) similar to the slope decrease (−33% ± 6%, *n* = 7) during the frequency-induced mode switch (see Figure 2D). This finding indicates that Na^+^ channel inactivation regulates bursting by reducing the initial slope of the ADP, an effect that could be mediated by persistent Na^+^ current, rapid reactivation of Na^+^ current, or current returning to the soma as the action potential propagates into the dendrites [20].

## Discussion

Identifying the specific mechanisms that govern hippocampal/subicular output is important for a greater understanding of cognitive and mnemonic functions (e.g., working memory and sleep), as well as pathological conditions (e.g., schizophrenia, addiction, epilepsy, and Alzheimer's disease) that have been linked to the subiculum [6,10,11,21–24]. Here we present evidence for a novel intrinsic mechanism that governs the output mode of bursting subicular neurons: a Na^+^ channel slow inactivation state.

In vivo and in vitro, we observed that subicular neurons generate bursts near the beginning of epochs of activity (less than 10 s) and later switch to single-spike firing. Induction of prolonged Na^+^ channel inactivation produces a frequency-dependent output-mode transition from bursting to single spiking by reducing the initial slope of the ADP in a burst. Thus, while we have shown previously that the ADP is mediated largely by a Ca^2+^ tail current [15], a critical component also depends on voltage-gated Na^+^ channels, thus rendering bursting susceptible to Na^+^ channel inactivation.

Slow (prolonged) inactivation of Na^+^ channels is distinct from conventional fast inactivation and has been studied in a number of preparations, including the squid giant axon; skeletal muscle; and neocortical, thalamic, and CA1 pyramidal neurons [17–19,25–27]. Despite numerous biophysical studies of slow inactivation, very little is known about its functional consequences at the cellular or network levels. We and others have previously suggested a role for slow inactivation of Na^+^ channels in regulating the amplitude of back-propagating action potentials in the dendrites of CA1 pyramidal neurons [17–19]. In bursting neurons of subiculum, it appears that the slow inactivation state of Na^+^ channels has a fundamental role in regulating pyramidal neuron output mode.

Transitions from bursting to single spiking have been observed in other cell types, such as thalamic relay cells and neocortical and hippocampal CA3 pyramidal cells. In the thalamus, sustained transitions from bursting to regular spiking are mediated by inactivation of T-type Ca^2+^ channels [13]. This is fundamentally different from the mechanism we describe here for pyramidal neurons of subiculum, both because the ion channels involved are different and because bursting is self-limiting in subiculum, but not in the thalamus (but see [28]). Self-limiting bursting has also been observed in pyramidal neurons of CA3 and neocortex [29,30]. Although the mechanisms have not been experimentally determined, transitions from bursting to regular spiking in CA3 have been modeled by activation of slow, Ca^2+^-activated K^+^ channels [31]. This mechanism is not responsible for the output-mode shift in subiculum, because transitions from bursting to single spiking routinely occurred at intervals longer than the burst or single-spike AHP (see Figure 2G). Furthermore, we found no evidence for activity-dependent increases in voltage-activated K^+^ currents (see Figure S4).

As a determinant of bursting or single spiking output in the subiculum, prolonged inactivation of Na^+^ channels is likely to be an important mechanism for regulating network activity. Neuromodulators that decrease slow Na^+^ channel inactivation would enhance the predominance of bursting, while increasing slow inactivation would reduce bursting in subiculum, thus changing the way the hippocampus activates its target structures. Recent work indicates that such modulation may indeed occur; in layer 5 neurons of the prefrontal cortex, serotonin receptors (5-HT_2_) modulate the slow-inactivation state of Na^+^ channels and can influence spiking dynamics [32]. Furthermore, it is possible that slow inactivation of Na^+^ channels is a general mechanism that conditionally regulates the output mode of bursting prefrontal cortex projection neurons.

Functionally, transient bursting followed by a shift to single spiking may serve to enhance the importance of new stimuli as they become represented within a network. For example, intrinsic bursting neurons are capable of converting brief, strong inputs into longer-lasting action potential outputs (bursts), which activate target structures more effectively than single spikes [4]. One major output pathway of subiculum is to the nucleus accumbens medium spiny neurons [33]. Hippocampal input to these neurons, via subiculum, quickly drives them from their very hyperpolarized (about −80 mV) “down” states to their more depolarized (about −55 mV) “up” states [34]. Subicular bursting may provide a “gating” signal at the beginning of the input, in order to drive target neurons into an activated “up” state. During sustained activity, bursting is gradually replaced by the weaker single-spike output mode, which may be sufficient to maintain the “up” state. By regulating spike-output mode in this way, prolonged inactivation of Na^+^ channels may be a key regulator of network function in the hippocampus and elsewhere in the brain.

## Materials and Methods

### 

#### Extracellular single-unit recording.

Male rats (42–50 d old) were anesthetized with chloral hydrate (400 mg/kg intraperitoneally) and mounted in a stereotaxic apparatus. The tail vein was catheterized to administer supplemental anesthetic. Body temperature was maintained at 36.5–37.0 °C. Glass electrodes were filled with a solution of 2 M NaCl and 1% fast green dye. The tip of the electrode was less than 2 μm in diameter (impedance 1.8–2.2 MΩ in 0.9% saline). The electrode was advanced through a small burr hole in the skull with a hydraulic microdrive (David Kopf Instruments, Tujunga, California, United States) to the dorsal subiculum. Single-unit and bursting extracellular waveforms were identified using a high-impedance amplifier (Fintronic, Foster City, California, United States), bandpass filtered (low cutoff, 400 Hz; high cutoff, 500 Hz), and monitored with an oscilloscope, computer, and audio monitor. An analog-to-digital interface (Digidata 1200; Axon Instruments, Foster City, California, United States) was attached to a computer running AxoScope 7.0 software. Bursting units and single-spiking waveforms were analyzed using a template-matching algorithm in SPIKE 2 (Cambridge Electronic Design, Cambridge, United Kingdom) and custom analysis macro written in IGOR Pro 5.01 (Wavemetrics, Portland, Oregon, United States). Event detection threshold was set at six standard deviations above the mean of the entire recording. Multiunit recordings were not included. Bursting neurons were identified by their high frequency (130–300 Hz) clusters of two to four action potentials, decreasing spike amplitudes with each burst, and broadening spike widths within each burst (initial spike widths = 1.2–1.8 ms).

#### Solutions and drugs

ACSF for slice experiments consisted of 125 mM NaCl, 25 mM glucose, 25 mM NaHCO_3_, 2.5 mM KCl, 1.25 mM NaH_2_PO_4_, 2 mM CaCl_2_, and 1 mM MgCl_2_ (pH 7.4) (bubbled with 5% CO_2_ and 95% O_2_). Kynurenic acid (2.5 mM), SR 95531 (2–4 μM), and atropine (1 μM) were added to the ACSF to block synaptic input. The whole-cell current-clamp recording solution contained 115 mM K-gluconate, 20 mM KCl, 10 mM Na_2_-phosphocreatine, 10 mM HEPES, 2 mM Mg-ATP, 0.3 mM Na-GTP, and 0.1% biocytin (pH 7.3). For nucleated voltage-clamp *I*_Na_ recordings, intracellular solutions were either CsCl-based (130 mM CsCl, 10 mM Na_2_-phosphocreatine, 10 mM HEPES, 2 mM EGTA, 2 mM Mg-ATP, and 0.3 mM Na_2_-GTP [pH 7.3]) or Cs-gluconate-based (115 mM Cs-gluconate with 20 mM CsCl substituted for 130 mM CsCl [pH 7.3]). Membrane potentials were not corrected for a −8-mV liquid junction potential. For cell-attached patch recording of *I*_Na_, the pipette solution contained 120 mM NaCl, 3 mM KCl, 10 mM HEPES, 2 mM CaCl_2_, 1 mM MgCl_2_, 30 mM tetraethyl-ammonium chloride (TEA), 5 mM 4-aminopyridine (4-AP) and NaOH (pH 7.4). For cell-attached patch recording of *I*_Ca_, the pipette solution contained 120 mM NaCl, 3 mM KCl, 10 mM HEPES, 5 mM CaCl_2_, 1 mM MgCl_2_, 30 mM TEA, 5 mM 4-AP, 0.1 mM nicotine, and 500 nM TTX (pH adjusted to 7.4 with NaOH). All drugs were obtained from Sigma (St. Louis, Missouri, United States).

#### Slice preparation****

Hippocampal slices were prepared from mature male Wistar rats (36–50 d old). Slices were incubated for 20–40 min in a chamber containing warm (34–35 °C) ACSF and maintained at room temperature until they were moved to the recording chamber (32–35 °C). For recording, slices were transferred individually to a chamber on a fixed stage of a Zeiss (Oberkochen, Germany) Axioscop equipped with DIC optics. Recordings were obtained with visualization Dage-MTI (Michigan City, Indiana, United States) tube camera.

#### Current-clamp recordings.

Whole-cell, current-clamp recordings were made from the soma using a BVC-700 amplifier (Dagan, Minneapolis, Minnesota, United States). Patch-clamp electrodes were fabricated from thick-walled borosilicate glass with resistances of 3–5 MΩ in ACSF. Synaptic stimulation of CA1 axons projecting to subiculum was performed using a bipolar stimulating electrode fabricated from theta glass and connected to a stimulus isolator (Axon Instruments, Union City, California, United States). Brief current injections were intended to mimic EPSCs by using a dual exponential function (τ_rise_ = 1 ms; τ_decay_ = 6 ms). Data were stored on a computer (Dell) via an ITC-16 analog-to-digital interface (Instrutech, Port Washington, New York, United States). Data acquisition and analysis was performed using Igor Pro. Group comparisons were made using paired or unpaired t-tests as appropriate.

#### Voltage-clamp recordings.

Cell-attached and nucleated outside-out patch recordings were obtained from slices prepared from 14- to 16-day old male rats using an EPC-7 amplifier (Heka Elektronik, Lambrecht/Pfalz, Germany). Nucleated-patch experiments were performed at room temperature for improved stability of nucleated patches and improved clamp of Na^+^ currents. Glass electrodes (3–5 MΩ) were coated with Sylgard. Nucleated patches were obtained with capacitance and leak subtraction as previously described [18]. Analysis was performed using averages of five to 20 sweeps.

#### Histological procedures

For in vivo experiments, the recording position was marked by local iontophoresis of fast green dye through the electrode. Electrode placement was verified using routine light microscopy from serial coronal brain sections (60 μm). For in vitro recording, the slices were placed in paraformaldehyde (4%) at 4 °C. We processed the biocytin-filled cells using an avidin, horseradish peroxidase, diaminobenzene reaction.

## Supporting Information

Figure S1 Power Spectral Density Plot of Local Field Potentials Recorded in the Subiculum In Vivo Note the predominant delta-frequency band (1–4 Hz) and a weaker theta-frequency band (5–10 Hz) recorded in vivo. The inset shows a 10-s sweep illustrating the local field potential fluctuations.
(8.5 MB TIF).

Figure S2 Transverse Section (300 μm Thick) of the Hippocampus Showing Paraformaldehyde-Fixed, Biocytin-Labeled Bursting Subicular Neuron (3.3 MB TIF).

Figure S3 In Vitro Current Clamp Recording of a Bursting Neuron in Response to a Noisy Current Stimulus Input Bursts (asterisks) transition to single spikes with sustained depolarization. The bottom trace shows the rate of rise *(dV/dt)* of the action potentials above. The *dV/dt* is high with each initial burst, decreases in size as the cell transitions to single spike mode, and recovers as the time between spike events increases.
(8.4 MB TIF).

Figure S4 Activity-Dependent Changes in Voltage-Gated K^+^ Current Cannot Explain the Transition from Bursting to Single Spiking The inset depicts sample K^+^ currents evoked by 2 ms depolarizations from −70 to 0 mV in cell-attached patches. Three superimposed traces are shown, each beginning with the response to two-step depolarizations (to mimic a burst) and followed by a single test response. Note that the K^+^ current depresses during the burst, but recovers rapidly and does not facilitate, as would be required to explain the activity-dependent output-mode transition. On the main graph, the normalized current amplitude in the test response is plotted as a function of recovery time after the burst (*n* = 6).
(10.4 MB TIF).

## Abbreviations

ADP: afterdepolarization
AHP: afterhyperpolarization
EPSP: excitatory postsynaptic potential
*I*_Na_: Na^+^ current
TTX: tetrodotoxin
sEPSC: simulated excitatory synaptic current
